# Altered Phase-Relationship between Peripheral Oscillators and Environmental Time in *Cry1* or *Cry2* Deficient Mouse Models for Early and Late Chronotypes

**DOI:** 10.1371/journal.pone.0083602

**Published:** 2013-12-26

**Authors:** Eugin Destici, Edwin H. Jacobs, Filippo Tamanini, Maarten Loos, Gijsbertus T. J. van der Horst, Małgorzata Oklejewicz

**Affiliations:** 1 Department of Genetics, Center for Biomedical Genetics, Erasmus University Medical Center, Rotterdam, The Netherlands; 2 Department of Molecular and Cellular Neurobiology, Center for Neurogenomics and Cognitive Research, Vrije Universiteit, Amsterdam, The Netherlands; University of Texas Southwestern Medical Center, United States of America

## Abstract

The mammalian circadian system is composed of a light-entrainable central clock in the suprachiasmatic nuclei (SCN) of the brain and peripheral clocks in virtually any other tissue. It allows the organism to optimally adjust metabolic, physiological and behavioral functions to the physiological needs it will have at specific time of the day. According to the resonance theory, such rhythms are only advantageous to an organism when in tune with the environment, which is illustrated by the adverse health effects originating from chronic circadian disruption by jetlag and shift work. Using short-period *Cry1* and long-period *Cry2* deficient mice as models for morningness and eveningness, respectively, we explored the effect of chronotype on the phase relationship between the central SCN clock and peripheral clocks in other organs. Whereas the behavioral activity patterns and circadian gene expression in the SCN of light-entrained *Cry1^-/-^* and *Cry2^-/-^* mice largely overlapped with that of wild type mice, expression of clock and clock controlled genes in liver, kidney, small intestine, and skin was shown to be markedly phase-advanced or phase-delayed, respectively. Likewise, circadian rhythms in urinary corticosterone were shown to display a significantly altered phase relationship similar to that of gene expression in peripheral tissues. We show that the daily dissonance between peripheral clocks and the environment did not affect the lifespan of *Cry1^-/-^* or *Cry2^-/-^* mice. Nonetheless, the phase-shifted peripheral clocks in light-entrained mice with morningness and eveningness-like phenotypes may have implications for personalized preventive and therapeutic (i.e. chronomodulation-based) health care for people with early and late chronotypes.

## Introduction

Circadian clocks provide an organism with a timing-mechanism to adjust a wide range of behavioral, physiological and metabolic processes to the specific time of the day and are found in diverse species, ranging from single-cell cyanobacteria and some fungi, to multi-cellular plants and animals [1]. These rhythms are thought to provide organisms with a survival advantage by allowing them to anticipate to daily changes in the environment [2]. Indeed, for cyanobacteria and plants it has been firmly established that strains in tune with the environment have better growth and survival characteristics [3], [4]. This was first postulated and tested in *Drosophila* by Pittendrigh who referred to this phenomenon as “circadian resonance” [5].

Circadian rhythms are generated by a molecular oscillator, which is composed of a set of clock genes that act through interlocked negative and positive transcription/translation feedback loops to drive cyclic expression of a set of clock genes with a period of approximately (*circa*) one day (*dies*) [6]. In short, the heterodimeric transcription activator CLOCK/BMAL1 activates transcription of the *Cryptochrome* (*Cry1* and *Cry2*) and *Period* (*Per1* and *Per2*) genes through E-box elements in their promoters. After being synthesized in the cytoplasm, CRY and PER proteins heterodimerize and subsequently enter the nucleus, where they inhibit CLOCK/BMAL1-mediated transcription, and thus shut down the expression of their own genes [6]. Likewise the CLOCK/BMAL1 heterodimer and CRY/PER complexes drive cyclic expression of clock-controlled genes (CCGs) that couple the molecular oscillator to rhythmic output processes. Amongst the CCGs are transcription factor genes (*e.g.* nuclear receptors [7]), cyclic expression of which allows E-box-independent circadian expression of output genes.

The central (“master”) circadian clock is housed in the neurons of the suprachiasmatic nucleus (SCN), which is located in the hypothalamus [8], [9]. To keep in phase with the light-dark cycle, the SCN receives light input from the environment, received by retinal photoreceptor cells and a subset of (melanopsin containing) ganglion cells, and delivered through the retinohypothalamic tract (RHT) [10]. Besides the SCN neurons, cells of almost all other tissues (including non-SCN brain tissue) have their own self-sustained circadian oscillator [11]. These peripheral clocks are synchronized by the SCN through humoral and neuronal signals [12], which act in a tissue-specific manner [13], and keep peripheral tissues in phase. Transcription profiling studies on SCN and peripheral tissues have shown that the circadian clock drives rhythmic expression of up to 10% of the transcriptome in a tissue-specific manner [14]–[17]. Tissue-specific inactivation of *Bmal1* in the liver and retina has shown that non-rhythmic expression of CCGs impacts on the physiology of these tissues [18], [19], thereby further illustrating the importance of peripheral circadian clocks. Moreover, disruption of the circadian system in rodent models either by genetic inactivation of clock genes or by enforcing chronic circadian dissonance (*i.e.* jetlag, protocols mimicking “shiftwork”), has been found to increase tumor growth, accelerate aging, and disrupt metabolism [20]–[22]. As such, it does not come as a surprise that epidemiological studies have provided evidence that disturbance of the human circadian clock due to *e.g.* repeated shift-work has been associated with a variety of pathologies such as cancer, metabolic syndrome and cardiovascular disease [20]–[22].

Whereas the inbred nature of rodent models used in laboratories causes minimal intra-animal variation, the human population is faced with a wide range of circadian periods, imposed by the marked genetic heterogeneity [23]. Analysis of the human chronotype (reflecting a person's tendency to be more active and alert early or late in the day) has revealed the existence of so called “larks” and “owls”, the morningness and eveningness phenotypes which are accompanied by differences in period length, amplitude and phase [24], [25]. Work, study and social obligations often force these individuals to wake up or stay awake together with their peers, and accordingly, to live out of phase with their internal clock. This situation is referred to as “social jetlag” [26] and opposes the situation encountered by shift workers and inter-continental airplane personnel. Rather than being faced with a genetically predisposed dissonance between body and environmental time, they “voluntarily” opt to temporarily live out of phase with their body clocks and environmental time. An even more dramatic dissonance between internal and external time is encountered in inherited autosomal dominant disorders known as Familial Advanced Sleep Phase Syndrome (FASPS) and Delayed Sleep Phase Syndromes (DSPS). Patients present dramatically shifted sleep-wake cycles and, unlike larks and owls, suffer from chronic fatigue and a strong tendency to develop depression [27]. FASPS mutations have been found in the human clock genes *PER2* and *CASEIN KINASE 1δ* [28], [29] and, when mimicked in the mouse, shortened the period of circadian behavior [29], [30].

Mice with inactivated *Cry1* or *Cry2* genes possess a circadian clock with a short (τ≈22.5 h) or long free-running period (τ≈24.6 h), respectively, as compared to control littermates (τ≈23.7 h) [31]. Since morningness and eveningness has been attributed to period length differences [24], [25], *Cry1* and *Cry2* knockout mice might be suitable animal models for exploring the consequences of these opposite phenotypes. Additionally, cultured cells and explanted tissues from these knockout mice display similar circadian phenotypes, although *in vitro* the difference in period length is more extreme [32], [33]. When maintained under a regular light-dark cycle (LD 12∶12), *Cry1^-/-^* and *Cry2^-/-^* mice entrain normally to the LD cycle [31], [34], except for a very small (∼10 min) phase advance in the onset of wheel running for *Cry1^-/-^* mice [34]. However, the performance of peripheral oscillators under regular LD cycles of *Cry1^-/-^* and *Cry2^-/-^* mice or other circadian mouse models with differences in intrinsic periods is not well characterized and survival consequences are lacking.

In the present study, we analyzed the clock- and clock controlled-gene expression in peripheral organs (*i.e.* liver, kidney, small intestines, skin) of *Cry* single knockout mice maintained under regular light-dark cycles. We show that whereas the phase of behavioral and SCN core oscillator rhythms are relatively normally entrained, peripheral clocks are markedly phase advanced and delayed in *Cry1^-/-^* and *Cry2^-/-^* animals, respectively. To start exploring the impact of shifted rhythms in peripheral organs, we determined here to what extent this daily internal phase dissonance in *Cry1^-/-^* and *Cry2^-/-^* mice affect their physiology and life span.

## Results

### Entrainment of *Cry1^-/-^* and *Cry2^-/-^* mice to the light-dark cycle

Given the recent reports showing that the phenotype of *Per1^-/-^* and *Per2^-/-^* mice is greatly diminished or even completely absent after backcrossing mice to a C57BL/6 background [30], [35], [36], we first analyzed the wheel-running behavior of C57BL/6JOlaHsd-backcrossed *Cry* mutant mice. Importantly, we found that in constant darkness (DD) *Cry1^-/-^* and *Cry2^-/-^* mice still displayed shorter and longer periodicity, respectively (*Cry1^-/-^*: 22.59±0.11 h; *Cry2^-/-^*: 24.19±0.10 h; wild type: 23.77±0.01 h), which compares very well with the circadian period of *Cry* mutant mice on a mixed 129Ola/C57BL/6JOlaHsd genetic background (*Cry1^-/-^*: 22.51±0.07 h; *Cry2^-/-^*: 24.63±0.061h; wild type: 23.77±0.07 h) as initially described [31].

**Figure 1:**
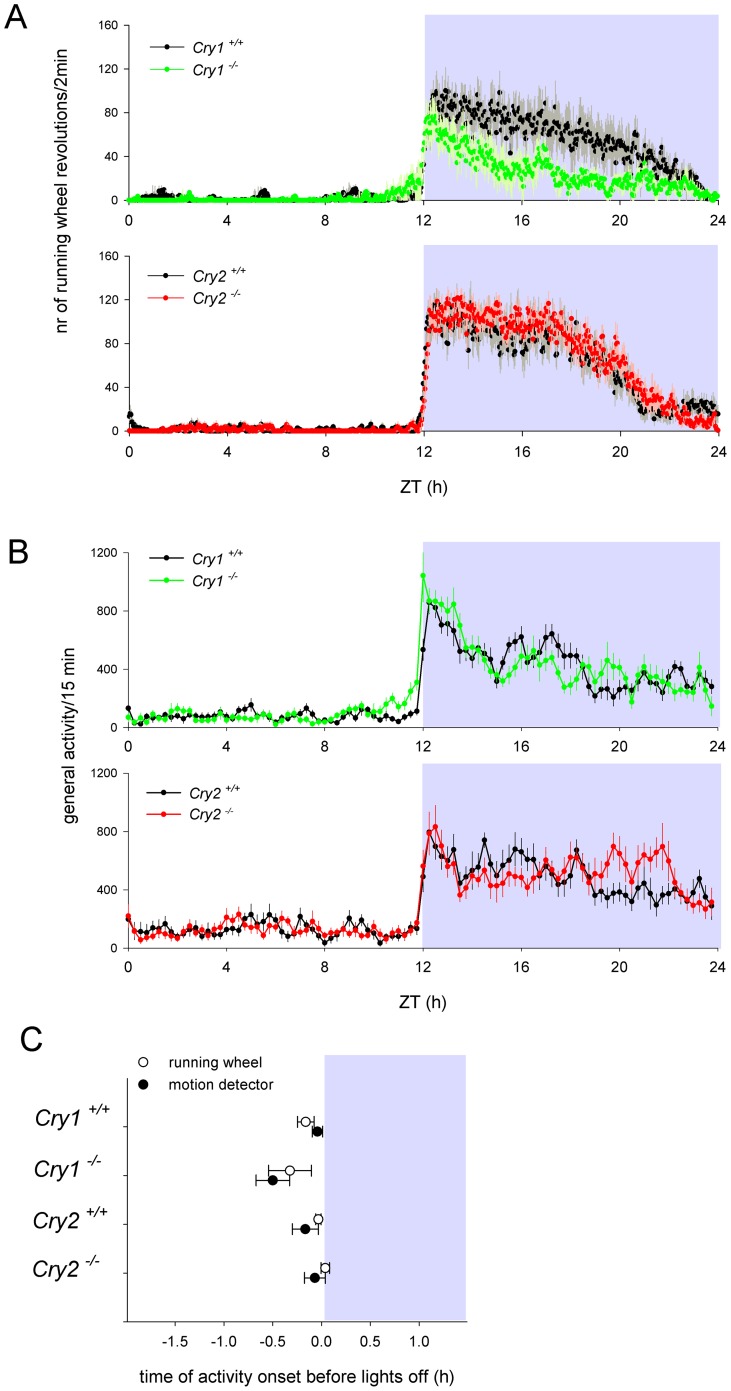
Locomotor activity of *Cry1^-/-^* and *Cry2^-/-^* mice. Five days average of daily distribution of locomotor activity of *Cry1^-/-^*, *Cry2^-/-^* and wild type littermate mice, as recorded in a running wheel set up (A) or by motion detectors (B). Upper and lower panels show activity distributions for *Cry1^-/-^* mice and their littermates and *Cry2^-/-^* mice and their littermates, respectively. (C) Average position of the onset of activity relative to the offset of light during exposure to LD 12:12 in *Cry1^-/-^*, *Cry2^-/-^* and their littermate mice recorded by running wheels (open symbols) and motion detectors (closed symbols). White and grey background indicates day and night, respectively. Error bars indicate standard error of the mean (s.e.m.).

When the mice were maintained under a regular 12 h light-12 h dark cycle (LD 12∶12), we found a small, but statistically not significant, advance in wheel-running activity onset of *Cry1^-/-^* mice (19.4±13.1 min; p>0.05), similar as has been reported by Spoelstra and co-authors [34]. Behavior of *Cry2^-/-^* mice was indistinguishable from that of their wild type litter mates (p>0.05; Figure 1A and C). Additionally, the *Cry1^-/-^* mice tended to be less active in the running wheels (Figure 1A). Since wheel-running behavior can be masked by light [37], the circadian behavior of mice was also analyzed using infrared video tracking (Figure 1B). The general locomotor activity data (Figure 1B and C) showed that the *Cry1^-/-^* mice became active earlier than the wild type mice (28.0±10.2 min; p<0.01). *Cry2* ^-/-^ mice did not start becoming active significantly later than wild type mice (5.1±6.2 min; p>0.1) and their level of activity was indistinguishable from that of wild type mice.

**Figure 2:**
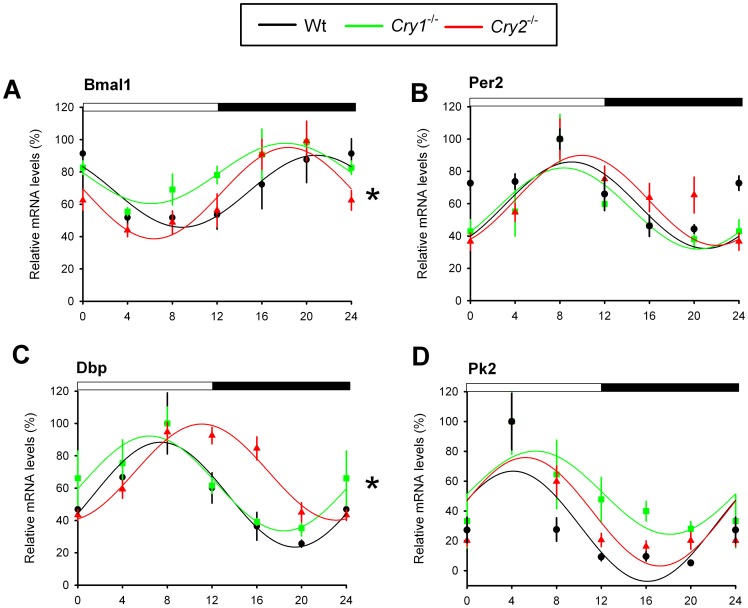
Circadian gene expression in the SCN of *Cry1^-/-^* and *Cry2^-/-^* mice. Relative mRNA expression profiles of core clock genes *Bmal1* (A) and *Per2* (B) and the clock-controlled genes *Dbp* (C) and *Pk2* (D) in the SCN of light entrained (LD) wild type (black), *Cry1^-/-^* (green) and *Cry2^-/-^* (red) mice, as measured by RT-qPCR. For each genotype, mRNA levels (mean ± s.e.m) are expressed relative to the peak of expression (set at 100%) and fitted with a wave curve. The two-way ANOVA test revealed a significant effect of genotype and time interaction (*p<0.05). Data at ZT0 and ZT24 are double-plotted for easier visualization of daily expression. The bar above panels indicates time of light on and off.

To test whether the relatively synchronous behavior of *Cry* single knockout mice is the direct result of a lack of phase differences in core clock performance in the central pacemaker, we analyzed circadian gene expression in the SCN from wild type, *Cry1^-/-^* and *Cry2^-/-^* mice maintained under a LD 12∶12 cycle. The expression of the clock genes *Bmal1* and *Per2* (Figure 2A and B) and the clock-controlled genes *Dbp* and *Pk2* (Figure 2C and D) were rhythmic across the genotypes (p<0.05). Overall, expression of *Bmal1* and *Dbp* over time was significantly different (p<0.05). However, the peak of expression for *Bmal1* coincided for *Cry1* ^-/-^ and *Cry2* ^-/-^ mice (the center of gravity was at ZT 18.1±3.4 h and ZT 18.4±3.0 h, respectively), which is earlier than observed in wild type mice (ZT 20.9±3.2 h). Furthermore, expression of *Per2* and *Pk2* (*Prokineticin* 2), a clock-controlled and light-inducible output gene that transmits circadian rhythm information from the SCN [38], had a similar mRNA expression pattern in all genotypes (p>0.05; Figure 2D). In conclusion, under light entrained conditions, differences in circadian period do not result in prominent phase shifts in diurnal behavioral and SCN clock gene rhythms.

### Characterization of peripheral circadian clocks in *Cry1^-/-^* and *Cry2^-/-^* mice

**Figure 3:**
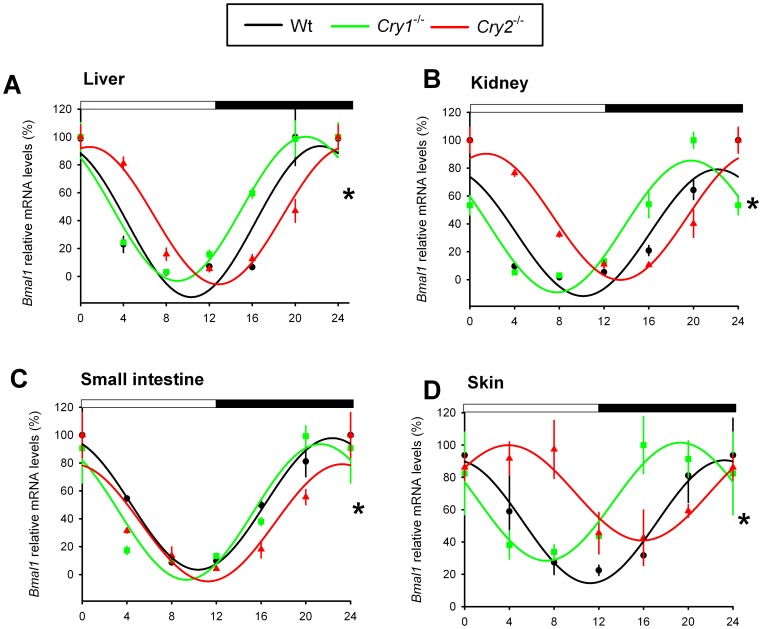
*Bmal1* expression profiles in peripheral tissues of light-entrained *Cry1^-/-^* and *Cry2^-/-^* mice. Relative mRNA expression profiles of *Bmal1* in the liver (A), kidney (B), small intestine (C), and skin (D) of light entrained (LD 12:12) wild type (black), *Cry1^-/-^* (green), and *Cry2^-/-^* (red) mice, as measured by RT-qPCR. For each genotype, mRNA levels (mean ± s.e.m) are expressed relative to the peak of expression (set at 100%) and fitted with a wave curve. The two-way ANOVA test revealed a significant effect of genotype and time interaction (*p<0.05). Data at ZT0 and ZT24 are double-plotted for easier visualization of daily expression. The bar above panels indicates time of light on and off.

**Figure 4:**
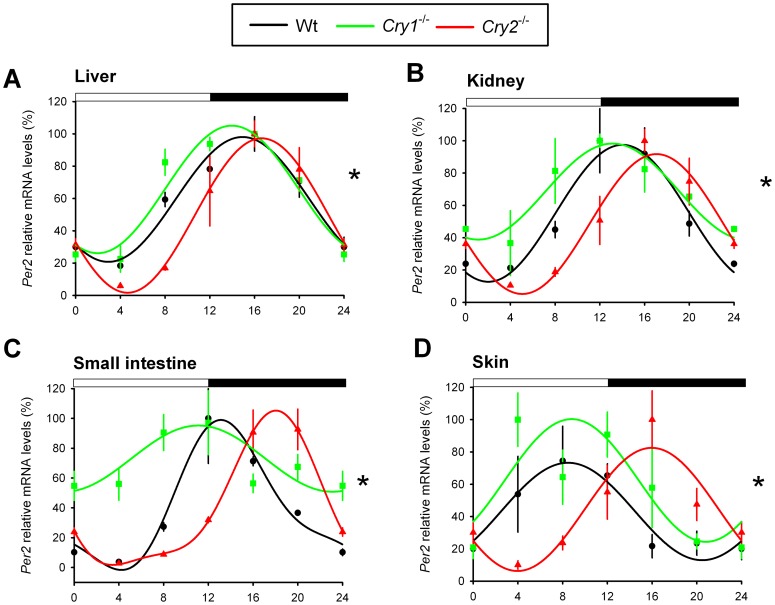
*Per2* expression profiles in peripheral tissues of light-entrained *Cry1^-/-^* and *Cry2^-/-^* mice. Relative mRNA expression profiles of *Per2* in the liver (A), kidney (B), small intestine (C), and skin (D) of light-entrained (LD 12:12) wild type (black), *Cry1^-/-^* (green) and *Cry2^-/-^* (red) mice, as measured by RT-qPCR. For each genotype, mRNA levels (mean ± s.e.m) are expressed relative to the peak of expression (set at 100%) and fitted with a wave curve. The two-way ANOVA test revealed a significant effect of genotype and time interaction (*p<0.05). Data at ZT0 and ZT24 are double-plotted for easier visualization of daily expression. The bar above panels indicates time of light on and off.

**Figure 5:**
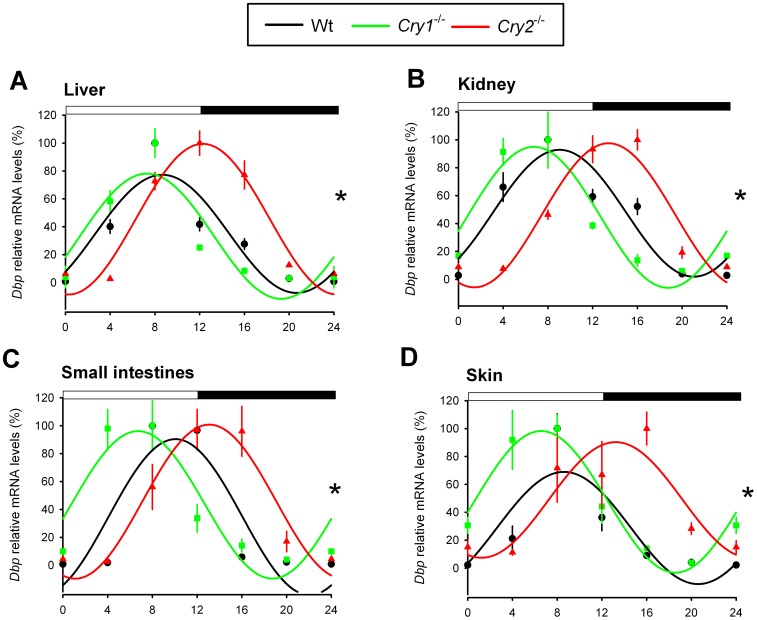
*Dbp* expression profiles in peripheral tissues of light-entrained *Cry1^-/-^* and *Cry2^-/-^* mice. Relative mRNA expression profiles of *Dbp* in the liver (A), kidney (B), small intestine (C), and skin (D) of light-entrained (LD 12:12) wild type (black), *Cry1^-/-^* (green) and *Cry2^-/-^* (red) mice, as measured by RT-qPCR. For each genotype, mRNA levels (mean ± s.e.m) are expressed relative to the peak of expression (set at 100%) and fitted with a wave curve. The two-way ANOVA test revealed a significant effect of genotype and time interaction (*p<0.05). Data at ZT0 and ZT24 are double-plotted for easier visualization of daily expression. The bar above panels indicates time of light on and off.

Next, we determined the phase of the circadian clock in several peripheral tissues from light-entrained (LD 12∶12) *Cry1^-/-^* and *Cry2^-/-^* mice *in vivo*, by analyzing the mRNA levels of the core clock genes *Bmal1* and *Per2* and the clock-controlled gene *Dbp* at 4 h intervals. Contrasting the entrained behavior and relatively synchronous SCN clocks, we found that *Bmal1* mRNA rhythms in the liver, kidney, small intestine, and skin of *Cry2* ^-/-^ mice were phase delayed, as compared to that of wild type mice (Figure 3). Moreover, in the *Cry1* ^-/-^ mice, *Bmal1* expression started to rise earlier in the liver, kidney, and skin (Figure 3A, B, and D). Similarly, *Per2* expression in the kidney, small intestine and skin started to rise earlier in the *Cry1^-/-^* and later in *Cry2* ^-/-^ mice as compared to wild type mice (Figure 4). In the liver, however, we did not observe marked phase differences in the peak for *Cry1* ^-/-^ compared with wild type mice (the center of gravity at ZT 14.4±2.8 h and ZT 14.9±2.6 h, respectively) and subsequent fall of *Per2* mRNA expression across genotypes. Yet, we observed a clear phase delay in the initial *Per2* mRNA accumulation in livers from *Cry2^-/-^* mice (Figure 4A). It has been shown that *Per2* is one of the 31 genes that are still cyclically expressed in the liver after hepatocyte-specific inactivation of the circadian clock in mice [39] and that cyclic expression of *Per2* is food driven [40]. Although there are differences in *Per2* mRNA accumulation in *Cry1^-/-^* and *Cry2^-/-^* livers during the inactive phase (ZT 0-12, Figure 4A), the regulation of *Per2* by food-driven transcription factors aligns the fall in expression of *Per2* in livers (ZT 12–24) from all three genotypes. Interestingly, the *Per2* expression profile in the kidney (Figure 4B) is in better agreement with the mRNA rhythm of the another CLOCK/BMAL1-target gene, *Dbp* (Figure 5), suggesting that the kidney contrasts the liver in that *Per2* oscillation is predominantly BMAL/CLOCK driven, rather than food driven.

The expression of *Dbp*, a well-characterized CLOCK/BMAL1-target gene involved in detoxification and drug metabolism [41]–[43], and thus a good readout for CLOCK/BMAL1 transcriptional activity, was phase shifted in *Cry* deficient mice in a tissue specific manner (Figure 5). In *Cry1^-/-^* mice, the *Dbp* peak timing in liver and kidney was normal, while the decline of expression appeared to be advanced (Figure 5A and B). In another study, in which *Dbp* expression in *Cry1^-/-^* livers was analyzed at 2 h intervals revealed a 2 h advance in *Dbp* peak expression [44]. It is possible that at a higher resolution of sampling (*e.g.* 2 h) we would be able to detect the difference in *Dbp* peak expression between wild type and *Cry1* ^-/-^ livers. In the small intestine, similar to *Per2, Dbp* expression showed a 4 h phase advance in *Cry1* ^-/-^ mice and 4 h phase delay in *Cry2* ^-/-^ mice (Figure 4C and 5C, respectively). In the skin, a tissue not expected to be affected by feeding behavior, we found very pronounced phase advanced and delayed rhythms in expression of all analyzed genes in *Cry1^-/-^* and *Cry2^-/-^* mice, respectively (Figure 3D, 4D, 5D).

Taken together, these data show that under regular LD 12∶12 conditions at least part of the circadian oscillator genes and the clock-controlled expression program in peripheral tissues of *Cry1^-/-^* and *Cry2^-/-^* mice are phase shifted, which contrasts the relatively synchronous central SCN clock and behavioral rhythms in these clock mutant mice.

### Physiological impact of internal dissonance in *Cry1^-/-^* and *Cry2^-/-^* mice

**Figure 6:**
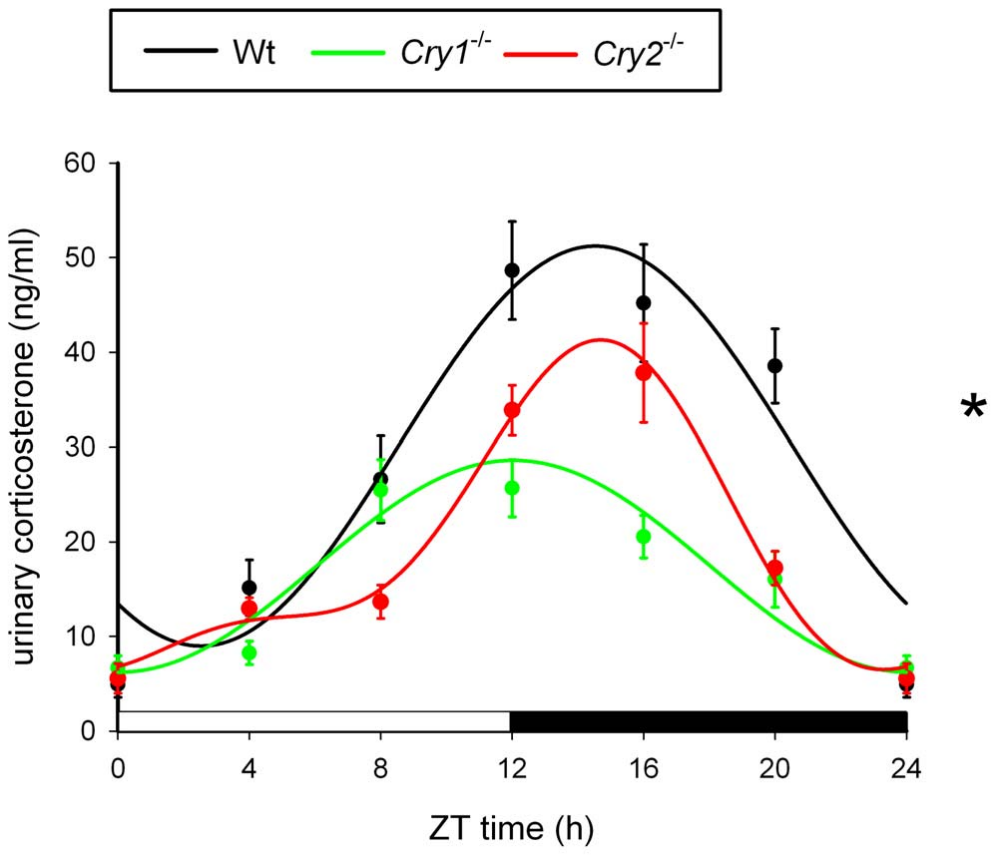
Circadian profiles of urinary corticosterone in *Cry1^-/-^* and *Cry2^-/-^* mice. Urine was collected at 4(black), *Cry1^-/-^* (green), and *Cry2^-/-^* (red) mice (± s.e.m; n = 8 per genotype). Data were fitted with a wave curve and analysis of variance revealed significant difference among genotypes (p<0.001). Data at ZT0 and ZT24 are double-plotted for easier visualization of daily expression. The bar below panel indicates lights on and off.

To determine the possible physiological consequences of the internal phase dissonance between peripheral oscillators of *Cry1* and *Cry2* deficient mice and the light-dark cycle, we assessed the phase of the daily corticosterone rhythm. Corticosterone, a glucocorticoid hormone secreted by the adrenal cortex, displays robust circadian oscillation in blood (unbound form) and urine (bound to globulin). We have chosen to analyze urine rather than plasma corticosterone as urine can be easily obtained in a non-invasive manner, which minimizes stress/handling-induced fluctuations of this stress responsive hormone [45]. As shown in Figure 6, the urinary concentration of corticosterone in wild type mice (measured at 4 h intervals over a 24 h period) displayed a clear circadian oscillation (p<0.001) and peaked around the onset of the dark phase (the overall center of gravity was at ZT 14.53±2.48 h), which is in agreement with recently published data [45]. Similarly, we could demonstrate significant circadian variation in urinary corticosterone levels between *Cry1* ^-/-^ and *Cry2* ^-/-^ mice (p<0.001) with peaks at ZT 12.04±2.59 h and ZT 14.12±2.36 h, respectively. Overall we found a significant effect of genotype (F(2,23) = 13.47, p<0.001) with a significantly phase advanced peak of corticosterone concentration in *Cry1* ^-/-^ mice as compared to *Cry2^-/-^* mice (p<0.001). However no differences were found between wild type and *Cry2* ^-/-^ mice (p>0.05). Taken together, these data demonstrate that in line with the shifted circadian gene expression in peripheral tissues, the urinary corticosterone rhythms are clearly phase advanced in *Cry1* ^-/-^ as compared to wild type and *Cry2* ^-/-^ mice.

**Figure 7:**
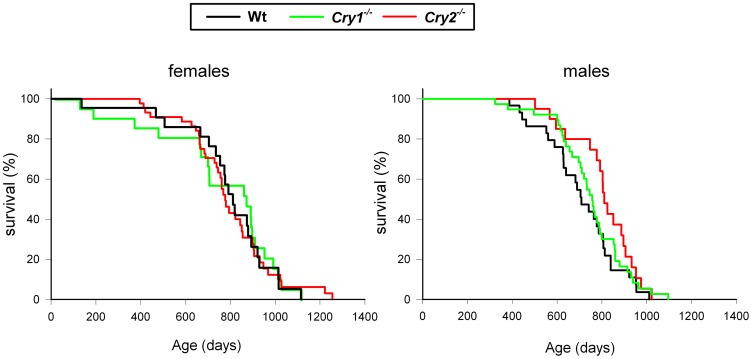
Life span of *Cry1^-/-^* and *Cry2^-/-^* mice. Age specific curves for female (left panel) and male (right panel) wild type, *Cry1^-/-^* and *Cry2^-/-^* mice. Animals were scored as dead from the cohort when found dead or when appearing moribund.

Next, we performed a large cohort study to compare the life span of *Cry1^-/-^* (n = 41), and *Cry2^-/-^* mice (n = 83) to that of wild type mice (n = 52). In addition, we monitored body weight and the development of pathological events. In line with our previous observation that *Cry1^-/-^* and *Cry2^-/-^* mice develop normally and do not display any overt pathology up to the age of 14 and 7 months, respectively [31], we did not observe any difference in body weight between wild type and *Cry* single knockout mice up to the age of one year (see Figure S1). Also during the remainder of their life, the mice did not develop any noticeable pathological phenotype (other than those also observed in aged wild type mice). The life span of females was shorter than that of males except for *Cry1^-/-^* females (Table 1). We tested the effect of sex, genotype, and the interaction between sex and genotype, on the rate of survival in a combined model using the Cox regression method. The sex difference, with overall male mice living slightly longer than females, as has been found before for a large cohort of C57BL/6J mice [46], was the only significant variable (W = 3.98, df = 1, p = 0.046; Figure 7) with no significant interaction between sex and genotype (W = 1.82, df = 2, p = 0.40). Likewise, genotype did not contribute to the explained variance in life span (W = 1.39, df = 2, p = 0.49; Figure 7). From these data, we conclude that advanced and delayed phase of peripheral oscillators in *Cry1^-/-^* and *Cry2^-/-^* mice do neither affect life span, nor trigger aberrant visible pathological events under regular 12 h light-12 h dark cycles.

**Table 1:** Characteristic of survival (in days) in male and female wild type (Wt), *Cry1* deficient (*Cry1^-/-^*), and *Cry2* deficient (*Cry2^-/-^*) mice.

Sex	genotype	n	mean survival (± s.e.m)	age at 50% survival	max survival (± sem)
Males	Wt	22	787 (47)	811	1018 (38)
	*Cry1^-/-^*	21	742 (59)	853	1018 (28)
	*Cry2^-/-^*	45	791 (31)	773	1017 (44)
					
Females	Wt	30	709 (31)	708	920 (28)
	*Cry1^-/-^*	20	807 (32)	810	971 (19)
	*Cry2^-/-^*	38	749 (26)	757	950 (27)

## Discussion

In the present study, we have provided evidence that in contrast to the central SCN clock, the peripheral circadian clocks as well as the expression pattern of clock-controlled output genes in peripheral tissues of short period *Cry1^-/-^* mice, housed under a regular light-dark regime (*i.e.* LD 12∶12) are phase advanced in comparison to that of wild type animals, while oppositely, these peripheral clocks are phase delayed in long period *Cry2^-/-^* mice in a tissue specific manner. The resulting dissonance between body time and environmental time likely mimics the condition encountered by human “larks” and “owls”, whose morningness and eveningness chronotype has been associated with alterations in the period and phase of their molecular circadian oscillator [46], [47]. Recent studies indicate that indeed the physiological period length of the human clock *in vivo* correlates with the period of the peripheral molecular oscillator in cultured fibroblasts from the same individuals [24], [25], [48]. Interestingly, the shifted melatonin profiles in early and late chronotypes are also accompanied by an altered phase of peripheral clock gene expression in oral mucosa [47].

### Phase difference in circadian gene expression in *Cry1^-/-^* and *Cry2^-/-^* tissues

Despite the near synchronous behavioral activity of *Cry1^-/-^* and *Cry2^-/-^* mice under a regular 12∶12 h LD cycle (as measured independently by wheel running activity and locomotor activity recordings), we found that the circadian expression of most clock and clock controlled genes in peripheral organs (liver, kidney, small intestine, and skin) is out of phase with the environment and with the circadian behavior of the mice.

However, we also observed that the phase of clock and clock controlled gene expression was not shifted uniformly across genotypes and peripheral tissues. Phases of gene expression were overall more delayed in *Cry2* deficient mice than advanced in *Cry1* deficient mice, as compared to the light-entrained behavioural activity rhythms. Furthermore, the phase of *Per2* mRNA peak and decrease in the liver of *Cry1* and *Cry2* deficient mice remains comparable to that of wild type animals. This is presumably due to the regulation of *Per2* expression by food-driven transcription factors in the liver [49], which apparently dominates over CLOCK/BMAL1-driven transcription. Interestingly, in contrast to the liver, the *Per2* mRNA rhythm in other food-entrainable tissues such as the intestine and kidney [49]–[51], remains phase uncoupled, suggesting that the stringency of food-driven *Per2* transcription is tissue-specific. The absence of large phase changes in *Per2* expression in the liver of the *Cry* deficient mice also implies that the feeding pattern of the mice is probably not phase-shifted, which is in line with the locomotor activity data. Conversely, the phase of *Bmal1* expression in kidney and skin was the most advanced and delayed in *Cry1* and *Cry2* deficient mice, respectively, as compared to wild type mice. These phase differences are likely responsible for the phase differences in the peak of CLOCK/BMAL1-driven transcription of *Per2* and *Dbp* in a tissues specific manner. Thus, the morningness and eveningness like phenotypes of *Cry1* and *Cry2* deficient mice, respectively, do not always translate into a similar phase difference of circadian gene expression rhythm in every peripheral tissue.

### Internal dissonance in *Cry1* or *Cry2* deficient mice and glucocorticoid concentration

We have shown that the altered phase of clock and clock-controlled genes in peripheral tissues of *Cry1* or *Cry2* deficient mice has an impact on the daily pattern of glucocorticoid release, as evident from the phase differences in urinary corticosterone rhythms especially in *Cry1^-/-^* mice. The mechanism of circadian corticosterone release is not fully understood but involves *i*) SCN-mediated circadian control of the hypothalamic-pituitary axis (HPA) via projections from the SCN to other parts of the central nervous system, including the pituitary gland, *ii*) SCN-mediated control through neural connections to the adrenal gland via the autonomic nervous system, and *iii*) local regulation of circadian oscillation by the clock machinery in the adrenal gland itself (for review see [52]). Since it has been reported that hypophysectomised animals still display a corticosterone rhythm [53], [54], one may exclude ACTH (adrenocorticotropic hormone produced by the pituitary gland) as a factor modulating corticosterone release. More likely, the observed phase alteration of the corticosterone rhythm in *Cry* deficient mice results from a direct or indirect neural signal from the SCN and an altered phase of the local clock in the adrenal gland. To obtain further insight into the altered phase of glucocorticoid secretion, it would be essential to examine not only the diurnal gene expression of clock genes in the pituitary gland and the adrenal cortex, but also secretion pattern of ACTH.

### Effect of internal dissonance on lifespan

In view of the resonance theory [5] and given the observation that (*i*) under regular 24 hour light-dark cycles mutant bacteria, plants, and hamsters with short or long period circadian clocks live shorter and perform worse than their wild type counterparts [3], [4], [55], [56] and (*ii*) chronic jetlag-mediated continuous body/environmental time dissonance has an adverse effect on the health of laboratory rodents [57], [58], we investigated whether the internal dissonance resulting from inactivation of *Cry1* or *Cry2* genes affects the life span of the mice under normal light-dark cycles. In contrast to our expectations, we did not find a significant impact of *Cry1* or *Cry2* deficiency on the life expectancy of mice, despite the fact that the peripheral clocks of *Cry1^-/-^*, and especially *Cry2^-/-^* mice, are continuously out of phase with the environment and behavior. Our findings also contrast the data obtained with heterozygote *tau* hamsters that display decreased survival under 12∶12 LD conditions [55], [56]. One explanation could be that heterozygote *tau* mutant hamsters with a free-running period of ∼22 h, unlike the *Cry1^-/-^* and *Cry2^-/-^* mice, display a dramatic phase difference of their activity onset (advanced by ∼4 h) when maintained in light-dark cycles [55]. Therefore, the period change in the *Cry* deficient mice and/or phase angle of entrainment are possibly not large enough to shorten their lifespan. Nevertheless, despite the normal behavior under LD, the phase difference in peripheral circadian gene expression in the *Cry1^-/-^* and *Cry2^-/-^* mice is profound.

Even though the internal circadian dissonance does not affect the life span of *Cry1^-/-^* and *Cry2^-/-^* mice under 12∶12 LD laboratory conditions, we do not exclude that a *Cry1* or *Cry2* deficiency could be disadvantageous under more competitive conditions in a more natural environment (*i.e.* photoperiod). Short versus long photoperiod and also different light-dark transitions (*i.e.* gradual twilight/dawn or rectangular) can affect the phase of the murine SCN oscillator [59], [60] as well as that of the murine liver clock [61]. In view of the intrinsic phase differences in peripheral clocks of *Cry1* and *Cry2* deficient mice, it could well be that these mice respond differently during the summer and winter, where differences between peripheral clocks and the environment can be expected to exert more pronounced effects.

Furthermore, it might be that *Cry1^-/-^* and *Cry2^-/-^* mice display other phenotypes, not necessarily connected with long-term survival. In this context it is interesting to note that functional magnetic resonance imaging of human “lark” and “owl” brains has revealed that maintaining attention in the evening was associated with higher activity in a few brain regions (including the SCN) in evening than in morning chronotypes [62]. Therefore, it would be of interest to measure gene expression in non-SCN brain regions (*e.g.* hippocampus [63]) of *Cry1^-/-^* and *Cry2^-/-^* mice under LD 12∶12 cycles to determine whether these peripheral (*i.e*. non-SCN) brain clocks are out of phase, similarly as observed for the peripheral clocks. If this is the case, the *Cry* deficient mouse models could additionally be used to mimic “larks” and “owls” in behavioral assays that address cognitive parameters (*e.g.* learning and memory).

In conclusion, we showed that under LD 12∶12 conditions, circadian gene expression in peripheral tissues of *Cry1^-/-^* and especially *Cry2^-/-^* mice has an altered phase-relationship with the central clock in the SCN, behavior and the environment leading to internal phase dissonance. This does not appear to affect the long-term survival of *Cry1^-/-^* or *Cry2^-/-^* mice but affects their hormonal rhythmicity and likely many other clock controlled output processes. In view of the minimal phase differences in the central clock of *Cry1* or *Cry2* deficient mice, we propose that the resetting signal from the SCN to the periphery is modulated by downstream endocrine organs (*e.g.* the pituitary gland, adrenal glands), resulting in phase shifted clocks in liver, kidney and other peripheral tissues of *Cry1^-/-^* and *Cry2^-/-^* mice. To date, there is a growing tendency to tailor therapeutic treatment protocols to the individual patient. An example of personalized medicine is the use of genetic information about a patient's tumor to facilitate the choice of the most effective anti-cancer drug. Likewise, proper timing of drug delivery (chronotherapy or chronomodulated therapy) has been shown to improve efficacy and reduce adverse side effects of drugs and is currently applied to diseases ranging from cancer and cardiovascular diseases to hypertension and psychiatric disorders [64]. Chronotherapy usually includes adjustment of drug delivery time on the basis of the patient chronotype, as determined by sleep questionnaires and sleep-wake recordings. However, the marked chronotype-dependent phase shifts in circadian rhythms in peripheral organs of mice with relatively synchronous behavior, as uncovered by this study, strongly point to a need to further personalization of chronotherapy by taking into account chronotype-dependent phase shifts in target organs. In this respect, the *Cry1* ^-/-^ and *Cry2* ^-/-^ mice represent an excellent tool to study the chronopharmacology and therapeutic efficacy of drugs in relation to morningness or eveningness chronotypes.

## Methods

### Ethics statement

Mice were kept at the Animal Resource Center (Erasmus University Medical Center), which operates in compliance with European guidelines (European Community 1986) and The Netherlands legislation for the protection of animals used for research, including ethical review. Animal studies at Erasmus University Medical Center were approved by DEC Consult, an independent Animal Ethical Committee (Dutch equivalent of the IACUC) under permit number 139-11-07 (EMC2383).

### *Cry1* ^-/-^ and *Cry2* ^-/-^ mice

The generation of *Cry1^-/-^* and *Cry2^-/-^* mice has been described previously [32]. Mice were backcrossed to C57BL/6JOlaHsd mice more than 9 times. Mice were housed at ambient temperature (19–22°C) and humidity (53–63%) under a 12 h light and 12 h dark cycle (LD 12∶12) with standard chow and water available *ad libitum* throughout the study.

### Assessment of circadian behavioral activity

Male *Cry* deficient mice (6 *Cry1* ^-/-^ and 6 wild type littermates and 8 *Cry2* ^-/-^ and 8 wild type littermates; age 8–10 weeks) were individually housed in type III polyester cages (Tecniplast, Italy), equipped with a steel running wheel (diameter 11.8 cm) connected to a sensor system to detect revolutions of the wheel. Voluntary wheel running (number of revolutions per 2 min) was continuously recorded using the ERS program (University of Groningen, The Netherlands). For general locomotor activity recordings 8–10 week old male mice (10 *Cry1* ^-/-^ and 12 wild type littermates and 11 *Cry2* ^-/-^ and 9 wild type littermates) were individually housed in a PhenoTyper (Noldus Information Technology, Wageningen, The Netherlands) equipped with infrared cameras and lights for video tracking (12.5 samples per second) using EthoVision 3.0 software. The distance moved spontaneously was determined in 15 min intervals. The light intensity in behavioral activity experiments ranged from 200 to 400 lx.

To assess entrainment to the LD cycle, the time of onset of activity was determined as the first bin where activity was greater than, or equal to 30% of peak activity on a specific 24 h time period and followed by three out of the next six bins having at least 30% peak activity. The phase angle of entrainment was defined as the number of minutes that activity onset preceded (+) or followed (−) the onset of darkness. The circadian period of running wheel activity was calculated over 12 full days of DD using ERS software, commencing one day after the start of DD (chi-square periodogram time series analysis).

### Quantitative RT-qPCR analysis

Tissues were isolated from light-entrained (LD 12∶12) wild type (n = 4 per time point), *Cry1^-/-^* (n = 3 per time point) and *Cry2^-/-^* (n = 3 per time point) mice, sacrificed at 4 h time intervals along the day (six time points). Animals were individually housed (age 12±2 weeks) in a room with a light intensity of 300±50 lx during the light phase of the LD cycle. Dissection of the brains during the dark phase of the cycle was performed under dim red light illumination. Liver, kidneys, hairless dorsal skin samples (∼2 cm^2^) and small intestines (around 2.5 cm) were isolated and snap-frozen in liquid nitrogen. For RNA isolation, liver and kidneys were powdered on dry-ice using a mortar and pestle. The powder was lysed in Trizol (Invitrogen), after which total RNA was isolated following a standard protocol (Invitrogen). Skin samples were homogenized using an Ultra-Turrax (IKA-Werke T25) in Trizol and intestines were homogenized in Trizol with TissueLyser II (Qiagen, 2 min at frequency 30/sec). The paired SCN was selectively dissected from 500 µm thick coronal section and stored in Lysis Buffer (Qiagen) at −80°C. RNA was purified with the Qiagen RNeasy Micro Kit (nr 74004) according to the manufactures protocol. For first-strand cDNA synthesis, 1 µg of RNA from peripheral tissues and 100 ng of RNA from the SCN was reverse transcribed using an iScript cDNA synthesis kit (Bio-Rad), following the manufacturer's protocol and subsequently 10 times diluted. Real-time qPCR analysis of the clock and clock-controlled genes was performed using SYBR green and an iCyclerIQ detection system (Bio-Rad). All samples were analyzed in triplicate. Primer sequences are available on request. Expression levels were normalized using *beta-2-microglobulin* (*β2m*) mRNA levels and plotted with the maximum expression level for each gene set at 100%. The generation of specific PCR products was confirmed by melting curve analysis and each primer pair was tested with a serial dilution of a cDNA mix that was used to calculate the primer pair efficiency.

### Life span study

To determine life span, a cohort of *Cry1^-/-^* mice (21 males, 20 females) and *Cry2^-/-^* mice (45 males, 38 females) was formed, along with a cohort of wild type control animals (22 males, 30 females). Mice were group housed with sex-matched littermates and kept under LD 12∶12 cycle with a light intensity of approximately 300 lx). The health state of the mice was checked daily, starting at the day of weaning. Individual mice were initially weighed weekly to determine body weight, and with longer intervals at later age. During the entire study, all mice were kept in the same and stringently controlled environment as described above. The microbiological status of the cohorts was monitored quarterly. Mice were scored as dead either when found dead, or when they had to be sacrificed according to the local bio-ethical standards (criteria applied: severe weight loss (>20%), no food-intake, no movement, no response to external stimuli and hunchbacked appearance).

### Corticosterone measurements

To determine urinary corticosterone levels, male mice (8 mice per genotype) were habituated to handling before urine collection. Mice were housed individually under LD cycle of 12 h light and 12 h dark with 300±50 lx illumination during the light phase. Urine sample collection was performed by placing a mouse on a parafilm sheet (Bemis ®) until it urinated. The mice that did not urinate spontaneously within one minute were picked up and restrained by the scruff and the base of the tail to force urination. Urine was then collected from the parafilm sheet with a pipette and immediately frozen on dry ice (−80°C). Urine collection was performed at 4 hour intervals. The corticosterone concentration was analyzed in duplicate with an ELISA kit (DRG Instruments, Marburg, Germany). The assay was optimized for use of urine samples (dilution 1∶4 with Standard 0). Average assay sensitivity was 0.29 ng/ml, mean recovery 97.3%, and intra- and inter-assay coefficients of variation were 3.1% and 11.1%, respectively.

### Statistical analysis

All data are presented as mean ± standard error unless mentioned otherwise. The activity onsets between genotypes for different method of activity recording were analyzed with the Mann-Whitney rank test. Differences in survival between groups were tested using the Cox regression method with Wald statistic (SPSS PASW 17). The rhythmic variation of gene expression and urinary corticosterone levels were tested by harmonic regression analysis using CircWave software (developed by R.A. Hut and available from http://www.euclock.org/). The average time (ZT) for peak of expression is presented ± circular standard deviation using CircWave software. The difference in gene expression among genotypes across all time points was analyzed with two-way ANOVA and the difference in center of gravity for corticosterone concentrations with one-way ANOVA (SigmaPlot 11). The Bonferroni t-test was used for *post-hoc* multiple comparisons. All tests were two-tailed with significance accepted at p<0.05.

## Supporting Information

Figure S1 **Body weight development of males (A) and females (B) wild type,** ***Cry1^-/-^*** **, and** ***Cry2^-/-^*** **mice.**
(TIF)
