# Supplementary figures and images for: Altered Phase-Relationship between Peripheral Oscillators and Environmental Time in Cry1 or Cry2 Deficient Mouse Models for Early and Late Chronotypes
Source: PLoS One. 2013 Dec 26;8(12):e83602. doi: 10.1371/journal.pone.0083602 (PMC3873389; doi:10.1371/journal.pone.0083602)

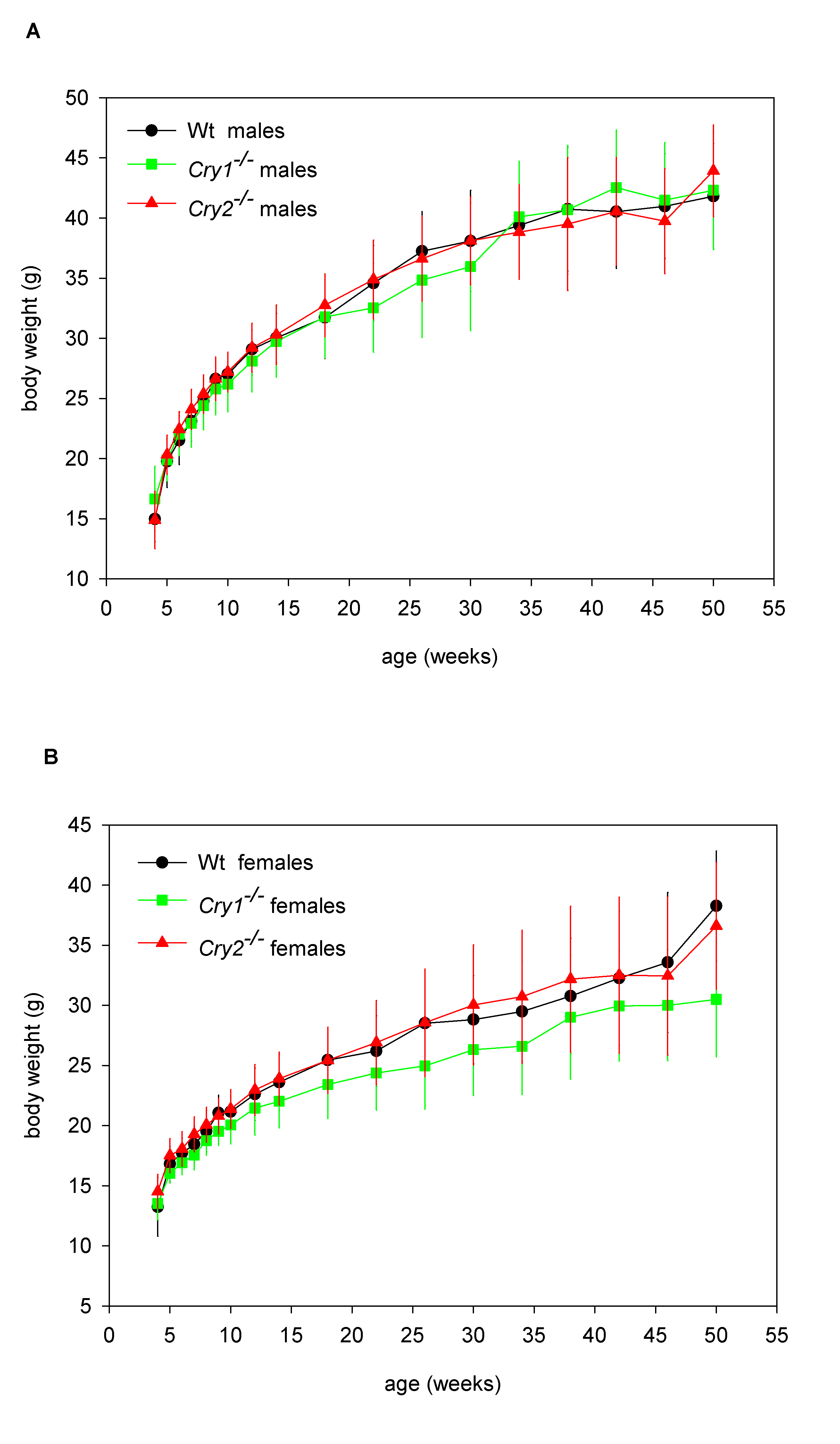

Supplement: Figure S1 — Body weight development of males (A) and females (B) wild type, Cry1-/- , and Cry2-/- mice. (TIF) [file pone.0083602.s001.tif]
